# Synthesis Characterization and Biological Activity Study of New Schiff and Mannich Bases and Some Metal Complexes Derived from Isatin and Dithiooxamide

**DOI:** 10.1155/2011/706262

**Published:** 2011-05-15

**Authors:** Ahlam J. Abdulghani, Nada M. Abbas

**Affiliations:** Department of Chemistry, College of Science, University of Baghdad, Jaderiya, Baghdad, Iraq

## Abstract

Two new Schiff and Mannich bases, namely, 1-Morpholinomethyl-3(1′ -N-dithiooxamide)iminoisatin (L_I_H) and 1-diphenylaminomethyl-3-1′-N-dithiooxamide)iminoisatin (L_II_H), were prepared from condensation reaction of new Schiff base 3-(1′-N-dithiooxamide)iminoisatin (SBH) with morpholine or diphenylamine respectively in presence of formaldehyde . The structures were characterized by IR, ^1^HNMR, mass spectrometry, and CHN analyses. Metal complexes of the two ligands were synthesized, and their structures were characterized by elemental analyses, atomic absorption, IR and UV-visible spectra, molar conductivity, and magnetic moment determination. All complexes showed octahedral geometries except palladium complexes which were square planar. The biological activity of the prepared compounds and some selected metal complexes was tested against three types of bacteria and against cell line of human epidermoid larynx carcinoma (Hep-2).

## 1. Introduction

**Scheme 1:**
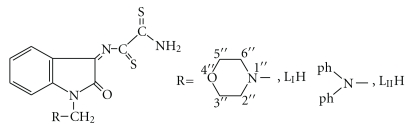
The structures of the prepared ligands.

Various Mannich Schiff bases of isatin have been found to be of biological importance [1] and have shown anticonvulsant [2], antibacterial [3, 4], antimicrobial [5–7] and anti-HIV activities [8, 9]. Dithiooxamide (dto) is an effective flexidentate complexing agent with varied coordination chemistry. Due to the intense chromophoric character, dto can be used in an imaging processes [10], coordination polymers [11], histological agents, and as a source for duplicating processes [12]. The transition metal complexes of dto and its derivatives are characterized by semiconductor, magnetic, and spectroscopic properties [12–15]. The aim of this work is to synthesize and study the coordination behavior of the two new Schiff and Mannich base ligands L_I_H and L_II_H shown in Scheme 1, from condensation reaction of a new Schiff base 3-(1′-N-dithiooxamide) iminoisatin (SBH) with morpholine or diphenylamine, respectively, in presence of formaldehyde in a mole ratio of (1 : 1 : 1), respectively, or from reaction of Mannich bases N-morpholinomethyl isatin (M_I_) and N-diphenylaminomethyl isatin (M_II_) [16] with dithiooxamide. The biological activity of the two ligands and some of their metal complexes was investigated against selected types of bacteria and against cancer cell line of human epidermoid larynx carcinoma (Hep-2).

## 2. Experimental/Materials and Methods

Melting points (uncorrected) were determined by using Gallenkamp MFB600–010f m.p apparatus. The purity of the synthesized compounds was checked by T.L.C. techniques using a mixture of chloroform and acetone (2 : 2 V/V) and various ratios of methyl acetate: acetone solvent mixture as eluents and iodine chamber for spot location. The HPLC of the Schiff base (SBH) and the derived two ligands were obtained by using HPLC (LKB), mobile phase CH_3_CN : H_2_O (80 : 20). Infrared spectra were recorded on a Perkin-Elmer 1310 IR spectrophotometer and Shimadzu corporation 200–91527 IR spectrophotometer using KBr and CsI disks. ^1^H n.m.r spectra of the organic compounds were recorded on a 300 MHz n.m.r spectrophotometer (Joel) using TMS as internal reference. Mass spectra were recorded on a Joel 700 mass spectrometer. Elemental CHN analyses were obtained by using EA elemental analyzer (Fison Ision Instrument). Electronic spectra of the ligands and their metal complexes in the region 200–1100 nm were recorded on a Shimadzu UV-visible-160 spectrophotometer. The metal contents were determined by atomic absorption technique using a Varian-AA-775 atomic absorption instrument. Electrical conductivity of metal complexes was measured at room temperature in DMF (10^−3^ M) using Elkta Lictfahigkeit conductivity meter (SIMENS). Magnetic moments (*μ*_eff_ BM) for the solid metal complexes at room temperature were determined according to Faraday's method by using Johnson Mattey magnetic balance system division. Chloride content of metal complexes was determined by potentiometric titration using 1686-titroprocessor-665 Dosinametrom (Swiss). All organic and inorganic materials were of high purity and used as received except ethanol, methanol, and DMF which were dried and distilled prior to use [17]. Palladium(II) chloride was converted to dichlorobis(benzonitrile)palladium(II) [18], and H_2_PtCl_6_·6H_2_O was converted to potassium hexachloroplatinate(IV) hexahydrate [19] prior to use. Mannich bases N-morpholinomethylisatin (M_I_) and N-diphenylaminomethyl isatin (M_II_ **)** were prepared according to methods mentioned in the literature [16]. Complex formation was studied in solutions to obtain the molar ratio of the ligand to metal ion (L : M) using ethanol, or DMSO as solvents. A series of solutions containing constant concentration of the metal ion (1 × 10^−4 ^M) were treated with various amounts of the same concentration of the ligand. The results of (L : M) ratio were obtained by plotting absorbance of solution mixtures at detected *λ*_max_ against [L]/[M]. 

## 3. Preparation of Ligands

### 3.1. 3-(1′-N-dithiooxamide)iminoisatin (SBH)

A solution mixture of isatin (0.01 mole, 1.47 g) and dto (0.01 mole, 1.021 g) in dry ethanol (50 mL) containing 2-3 drops glacial acetic acid was heated under reflux for 8 h with continuous stirring. The mixture was then left at room temperature for 24 hs. A yellow precipitate was formed. The product was filtered, washed with warm ethanol, and crystallized from ethanol:dichloromethane solvent mixture (1 : 1). m.p. 190°C yield 60%; IR (KBr) *ν*(cm^−1^): 3296, 3203 (NH_2_); 3147 (NH-isatin); 1733 (C=O), 1614 (−C=N); 1540 (C–S + *δ*NH, I); 1430 (C–N + C–S, II); 1197 (C–S, III); 835 (C=S, IV). ^1^H n.m.r. *δ*(ppm) (CD_2_Cl_2_) 12.012 (1H, s, NH); 7.653–6.94 (4H, m, aromatic); 2.022 (2H, d, NH_2_). MS, (m/z) (I%) (EI) calculated for C_10_H_7_N_3_OS_2_ m.wt 249 g/mole: 250 (10) [M+1]; 221 (3.2) [M–CO]; 207 (4) [M–NCO]; 162 (2); 119 (24); 90 (7.5). 

### 3.2. 1-Morpholinomethyl-3-(1′-N-dithiooxamide)iminoisatin (L_I_H) and 1-diphenylaminomethyl-3-1′-N-dithiooxamide)Iminoisatin (L_II_H)

(a) To a stirred solution of 3-(1′-N-dithiooxamide)iminoisatin (SBH) (0.01 mole, 2.49 g) and formaldehyde 37% (0.015 mole) in warm dry ethanol (20 mL) was added, drop by drop, (0.01 mole) of morpholine (L_I_H) or diphenylamine (L_II_H). The mixture was heated under reflux for 3 h with continuous stirring, and then left to cool at room temperature. A solid precipitate was formed. The products were filtered, washed with warm ethanol, and then crystallized from ethanol : chloroform (1 : 1 v/v) mixture; yield 35 and 28.2%, respectively.

(b) To a solution of dto (5 mmole, 0.6 g) in warm ethanol (10 mL) containing 2-3 drops glacial acetic acid was added (5 mmole) of Mannich base N-Morpholinomethylisatin (M_I_) or N-Diphenylaminomethyl isatin (M_II_) [16] in ethanol (10 mL) with continuous stirring, and the mixture was heated under reflux for 10 h. After leaving the mixture at room temperature for 24 h a precipitate was formed. The products were filtered, washed with warm ethanol, and crystallized; yield 20.3 and 21%, respectively (m.p. 215 and 283°C, resp.).

***1-Morpholinomethyle-3-*(1′*-N-dithiooxamide*)*iminoisa- tin* (L_I_H):**

yellowish orange crystals, m.p. 162°C; IR (KBr). *ν*(cm^−1^): 3203, 3138 (NH_2_); 3030–3000 (arom CH); 2815–2364 (CH_2_); 1730 (C=O); 1614 (−C=N); 1589 (C=C arom.); 1540 (*ν*C=N, *δ*NH, I); 1429 (C–N + C–S, II); 1195 (C–S, III); 835 (C=S, IV); 1149, 1328 (morpholine). ^1^H n.m.r *δ*(ppm) (DMSO): 7.59-6.898 (4H, m, aromatic +NH); 4.09 (2H, d, N–CH_2_N); 3.65 (4H, d, 2^”^, 6^”^ CH_2_ morph.); 2.33 (4H, d, 3^”^, 5^”^ CH_2_ morph.); 1.567 (1H, br, SH). MS (FAB) m/z (I%) calculated for C_15_H_16_N_4_O_2_S_2_, m.wt 348.45 g/mole,: 349.1 (93) [M]; 320 (38) [M–CO]; 235 (100); 234 (80); 220 (15), 207 (40). 131 (43); 104 (78%); (EI) m/z (I%): 348.5 (84) [M]; 320 (25) [M–CO]; 234 (38); 207 (17); 130 (19); 117 (24); 104 (57); 90 (30); 78 (22%). CHN% Calculated for C_15_H_16_N_4_O_2_S_2_ C, 51.67; H, 4.59, N, 16.07% found C, 50.69; H, 4.20; N, 15.75%; *ν*_max_ (cm^−1^) (DMF) (*ε*_max_ mol^−1^ cm^−1^) 34013 (20030) *π* → π*; 2415 (2980), *n* → π* (DMSO) 38461 (19519) *π* → π*.

***Diphenylaminomethyl-3-*(1′*-N-dithiooxamide*)*Iminoisa tin* (L_II_H):**

yellow red crystals, m.p. 182, IR (KBr) *ν*(cm^−1^): 3193, 3034 (NH_2_); 1730 (C=O); 1614 (C=N); 1540, 1434, 1195, 833 (C–N + *δ*NH, C–N + C–S, C–S, C=S I–IV, resp.). ^1^H n.m.r (*δ*, ppm) (CD_2_Cl_2_): 7.59–6.89 (14H, m, aromatic); 4.83 (2H, d, CH_2_); 2.17 (2H, b, NH_2_). MS m/z (I%) (EI): calculated for C_23_H_18_N_4_OS_2_, m.wt 430.55 g/mol: 430.9 (5.5) [M]; 402 (2.5) [M–CO]; 235 (21); 129.2 (1.8); 104 (4.8); 89 (10.3); 78.1 (3.5%). CHN (%) calculated for C_23_H_18_N_4_OS_2_: C, 64.18, H, 4.21; N, 13.02% found: C, 64.02; H, 4.22; N, 13.52%. *ν*_max_ (cm^−1^) (DMF) (*ε*_max_, L mol^−1^ cm^−1^) 32258 (22970) *π* → π*; 24509 (3530) *n* → π*; (DMSO): 38461 (19540): 28571 (14120) *π* → π*.

## 4. Synthesis of Metal Complexes

To a solution of Schiff Mannich base ligand (2 mmole) in absolute ethanol (L_I_H) or ethanol and dimethylsulfoxide (1 : 1 v/v) (L_II_H) (5 mL) was added an alcoholic solution (5 mL) of the metal salt (chlorides, nitrates, or acetates) (1 mmol), and the mixture was heated under reflux with continuous stirring for 3 h. Precipitation of products took place after heating time of 30 min for (Co(II)), Ni(II), and complexes of L_I_H and L_II_H (C_2_, C_3_, C_8_, and C_9_, resp.), 1 h for (Mn(II), Cu(II), and Ir(III) complex of L_**I**_H and Cd(II) complex of L_II_H (C_1_, C_4_, C_5_, and C_12_, resp.), 1.5 h for Pt(IV) complex of L_I_H (C_7_), 2 h for Pd(II) complex of L_I_H and Pt(IV) complex of L_II_H (C_6_ and C_11_, resp.) and 3 h for Pd(II) complex of L_II_H (C_10_). The products were filtered and purified from reactants by washing many times with ethanol and ether (C_1_–C_7_) or with DMSO, ethanol and ether (C_8_–C_12_), and vacuum dried. Purity of the products was detected by TLC, using silica gel as a stationary phase and a mixture of chloroform and acetone (2 : 2 V/V) or various ratios of methyl acetate: acetone solvent mixture as eluents.

## 5. Biological Activity Study

### 5.1. Antibacterial Action

Antibacterial activities of the prepared compounds were tested against three types of pathogenic bacteria, namely, *Escherichia coli*, *Staphylococcus areus,* and *Proteus mirabilis* using the antibiotic Ceftriaxone as a control. Bacterial cultures were prepared by streaking (0.1) mL of 10^6^ CFU/mL broth of indicator strain on the whole surface of nutrient agar plate. In each plate four wells (pores) were created on the nutrient agar layer using sterile cork porer. In each hole was injected 50 *μ*L of 10^−3^ M of the studied compounds in DMSO by micropipette. The resulting cultures were incubated at 37°C for 24 h. The inhibition zones caused by each compound were measured, and the results were interpreted according to diameter measurements.

### 5.2. Cytotoxic Activity

A preliminary study of cytotoxic activity of some of the prepared compounds was performed against human epidermoid larynx carcinoma cell lines (Hep-2) of 52-year-old patient. Hep-2 monolayer cell lines were prepared by subculturing cell line into (RPMI-1640) medium supplemented with 10% heat deactivated fetal bovine serum. The resulting media were incubated at 37°C for 48 h until confluent layer was achieved. Four concentrations of investigated compounds were prepared: 62.5, 125, 250, and 500 *μ*g/mL using dimethyl sulfoxide (DMSO) as a diluent. Hep-2 cell line was plated into 96-well microtiter plates. Then 0.2 mL of each tested compound was added to each well in triplicates, and incubation was carried out for 48 h. Cultures were stained with 50 *μ*L/well Neutral Red (NR) solution. The stained cultures were left in the incubator for further 2 h, washed with phosphate buffered saline solution followed by (0.1 mL) ethanol phosphate buffered solution (NaH_2_PO_4_ : ethanol (1 : 1), vehicle ethanol). The cytotoxic effects of the applied compounds were measured in terms of optical density of viable cells at *λ* = 492 nm using a Micro ELISA reader. 

## 6. Results and Discussions

### 6.1. Synthesis

**Scheme 2:**
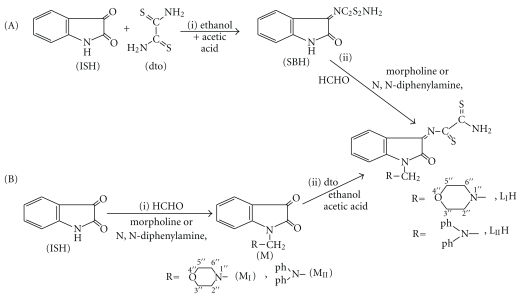
Synthesis of Schiff Mannich base ligands from isatin and dithiooxamide.

The synthesis of the two new ligands has been achieved by following two different pathways A and B as is illustrated by Scheme 2. Pathway A involves the synthesis of Schiff base precursor of isatin (SBH) followed by condensation with the secondary amine, morpholine or diphenylamine, in presence of formaldehyde to form L_I_H and L_II_H, respectively. Pathway B involves the formation of Mannich base precursor of isatin (M_I_ and M_II_) followed by condensation reaction with dithiooxamide. The second method showed lower yield and longer reaction time. 

**Figure 1:**
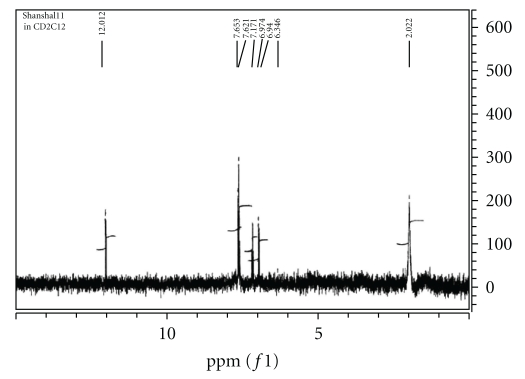
^1^HNMR Spectrum of SBH in CD_2_Cl_2_.

**Figure 2:**
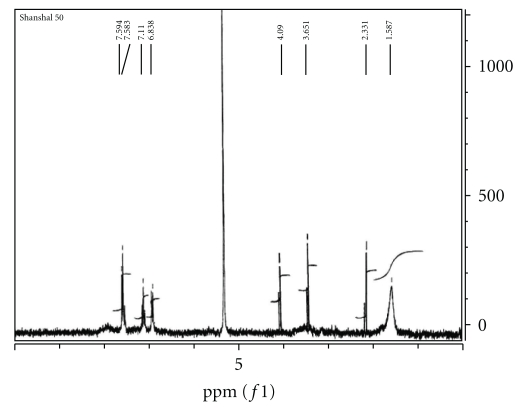
^1^HNMR Spectrum of L_I_H in DMSO.

**Figure 3:**
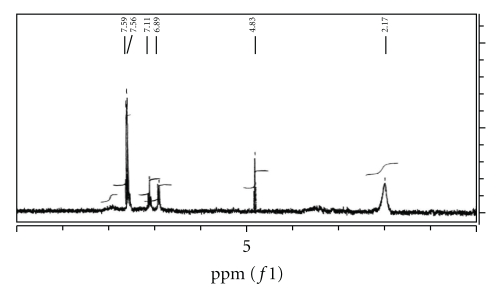
^1^HNMR of L_II_H spectrum in CD_2_Cl_2_.

The ^1^H n.m.r spectrum of the Schiff base precursor SBH in CD_2_Cl_2_ (Figure 1) is characterized by the appearance of chemical shift related to the NH_2_ protons of dto moiety at *δ*2.022 ppm [11, 20, 21] and the appearance of NH proton of isatin ring at *δ*12.012 ppm [5–7, 22–25] which is quite agreeable with the suggested structure of SBH. The ^1^H n.m.r spectrum of L_II_H in CD_2_Cl_2_ exhibited chemical shifts of NH_2_ protons at 2.17 ppm while that of L_I_H in DMSO (Figure 2) gave chemical shifts at *δ*1.567 ppm. This was attributed to tautomerism of L_I_H in DMSO to iminosulfhydryl structure in equilibrium with dithioamide structure, as a result of solvent polarity [26, 27]. Such behavior was confirmed by the appearance of the signal assigned to imino NH group at lower field. The spectrum of L_II_H (Figure 3) exhibited chemical shifts of aromatic protons of isatin ring and diphenylamine at *δ*6.89–7.11 and at *δ*7.68–7.59, respectively, while those of methylene group appeared at high fields [22–24].

**Figure 4:**
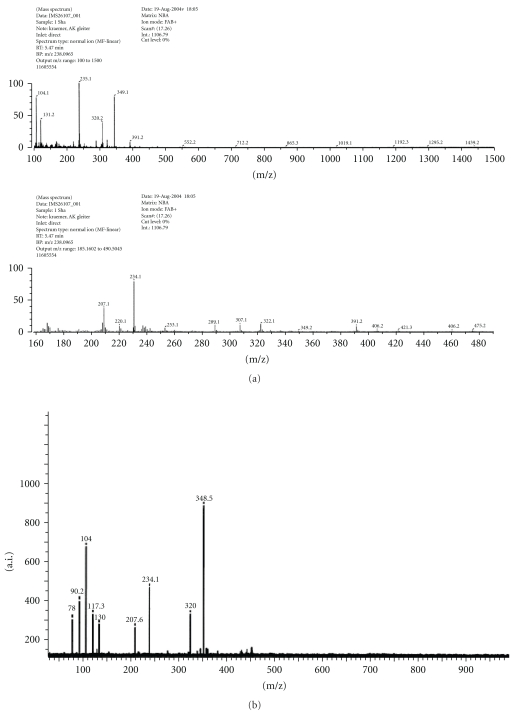
Mass spectrum of L_I_H by (a) FAB and (b) EI modes.

**Figure 5:**
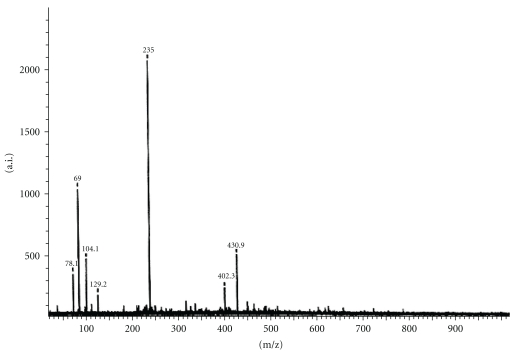
Mass spectrum of L_II_H.

**Figure 6:**
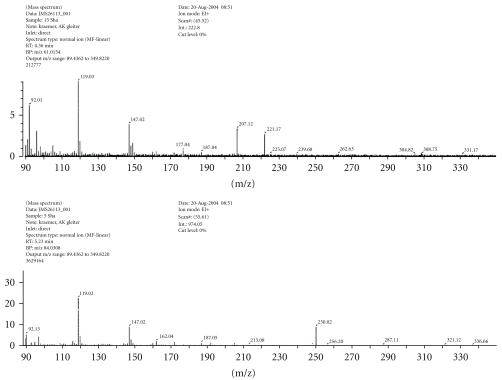
Mass spectrum of SBH.

The mass spectra of the two Mannich and Schiff base ligands as well as SBH are shown in Figures 4, 5, and 6, respectively. The EI mode of mass spectrum displayed by SBH (Figure 6) gave a peak at m/z = 250 which was assigned to [M+1], while the two Mannich base ligands displayed peaks corresponding to [M^+^] molecular ions. Smaller fragments were also observed and were characteristic of isatin behavior of other compounds [1, 22, 26, 28–34]. The FAB and EI modes of L_I_H (Figures 4(a) and 4(b)) showed different intensities of common fragments ions.

The IR spectra of the three organic compounds exhibited the disappearance of stretching modes assigned to C-3 carbonyl of isatin ring and appearance of stretching modes of azomethine group of Schiff base products at 1614 cm^−1^ [35]. Stretching vibrations of C-2 carbonyl group of isatin ring for SBH and the two Mannich Schiff base ligands were observed at 1733–1730 cm^−1^  [35]. The presence of bands assigned to NH_2_ asymmetric symmetric stretching vibrations indicates that the formation of Schiff bases was through one NH_2_ group only. Both ligands exhibited the absence of stretching vibrations assigned to NH of isatin ring, and instead vibrational modes of N–CH_2_ groups were observed at 2813–2304 cm^−1^ [35]. Bands observed at 1149, 1328 in the spectrum of L_I_H were attributed to C–O–C and C–N–C vibration of morpholine ring, respectively [35–38].

### 6.2. Physical Properties and Analytical Data of Metal Complexes

The color, melting points, yields, and elemental analyses of the prepared metal complexes of isatin Schiff Mannich base ligands are described in Table 1. Most results were in agreement with the suggested formula. Some deviations in elemental analyses may be attributed to incomplete combustion of the complexes. The low yield resulted from extensive purification of products from the starting materials as was indicated from TLC results.

### 6.3. Infrared Spectra

The important stretching vibrations of L_I_H and L_II_H metal complexes are described in Table 2. The Mn(II), Co(II) and Ni(II) complexes of L_I_H (C_1_–C_3_, resp.) and Co(II) complex of L_II_H (C_8_) exhibited shifts of the thioamide groups to lower frequencies indicating the involvement of thiocarbonyl sulfur atoms in coordination with these metal ions [39, 40]. The spectra of C_1_ and C_2_ demonstrated further shift of NH_2_ group vibrational modes to lower frequencies as a result of bonding. On the other hand the spectra of Cu(II), Ir(III), and Pt(IV) complexes of L_I_H (C_4_, C_6_, C_7_, resp.) and Pd(II), Pt(IV), and Cd(II) complexes of L_II_H (C_10_–C_12_, resp.) displayed the disappearance of the stretching mode of thioamide NH_2_ group and the shift of C-S band to lower frequencies. This refers to the bonding of metal ion to the deprotonated group of the ligand in the form of 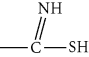 as in C_4_, C_6_, and C_9_ or in the form of 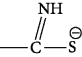 anion as in the case of C_5_, C_7,_ and C_10_–C_12_. The appearance of stretching modes assigned to NH and C=N of −C=NH groups was observed at 3371–3100 and 1640–1620 cm^−1^, respectively [15, 35, 39, 41]. The stretching vibrations of azomethine group of the Schiff base ligands were shifted to lower frequencies in all spectra except those of C_6_, C_10_, and C_12_, whereas stretching vibrations of carbonyl group were shifted to lower frequencies in all spectra except C_1_, C_4_, C_6_, and C_10_ indicating additional coordination of metal ions to C=N and C=O groups [36–38]. Bands related to coordinated water vibrations were observed in the spectra of C_1_, C_4_, and C_12_ at (3490, 756, 640), (3480, 800, 730), and (3500, 864, 710) cm^−1^, respectively, and to lattice water vibrations at frequency range 3519–3464 cm^−1^ in the other complexes. The bands related to nitrate ions were observed in the spectra of C_2 _and C_3_ at (1522, 1478), (1765, 1641) cm^−1^ and were assigned to monodentate and free ion behaviors, respectively, whereas that of C_8 _appeared at 1750–1660 and 1406–1380 cm^−1^ showing monodentate and bidentate behaviors, respectively [42]. Bands attributed to acetate group vibrations were observed in the spectra of C_9_ and C_12_ at (1645, 1340) and (1590, 1465) cm^−1^, respectively, indicating monodentate and bidentate bridging behaviors, respectively [42]. Additional bands were observed at lower frequencies (600–250 cm^−1^) and were attributed to M–N, M–O, M–S, and M–X (X = acetate, NO_3_^−^, Cl^−^) stretching modes [42].

### 6.4. Thermal Analysis

**Figure 7:**
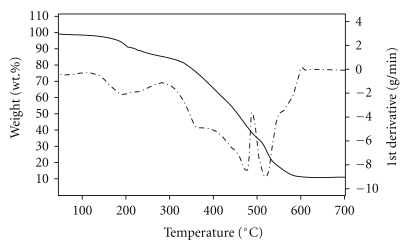
Thermographs of the Co(II) complex of L_I_H (C_2_).

**Figure 8:**
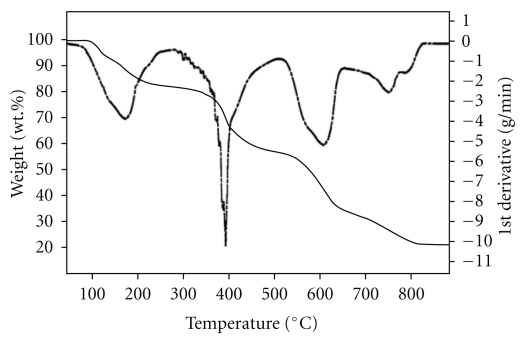
Thermographs of the Ir(III**) **complex of L_I_H (C_6_).

Steps of thermal decomposition of the Co(II) and Ir(III) complexes of L_I_H (C_2_, C_6_) following TG and DTG curves under nitrogen atmosphere and heating range 50–800°C are described in Table 3, and their thermographs are shown in Figures 7 and 8, respectively. At low temperatures, the initial weight losses were determined from TG curves referred to loss of water of crystallization [43–45]. The final stage of thermal decomposition of C_2_ gave the metal oxide whereas the Ir complex (C_6_) gave the free metal as a final residue [43–45].

### 6.5. Electronic Spectra and Suggested Structures

Table 4 describes the energies of bands observed in the spectra of metal complexes and their assignments together with magnetic moments and molar conductivity in DMF (10^−3^ M). The spectral parameters 10 Dq, $\text{Dq}/\bar{B}$, $\bar{B}$, and *β* as well as energies of unobserved ligand field bands were obtained by applying observed band energies and band ratios on Tanabe-Saugano diagrams of the specified metal ion [46–48]. All metal complexes exhibited spectra related to octahedral arrangement of ligand atoms around the metal ions except those of palladium(II) as they gave square planar geometries. The high values of magnetic moments of Co(II), Ni(II), and Cu(II) complexes are attributed to spin-orbital coupling [49]. All complexes were of high-spin octahedral geometries except Pt(IV), Ir(III), and Cd(II) complexes which were diamagnetic and so were Pd(II) complexes.

**Scheme 3:**
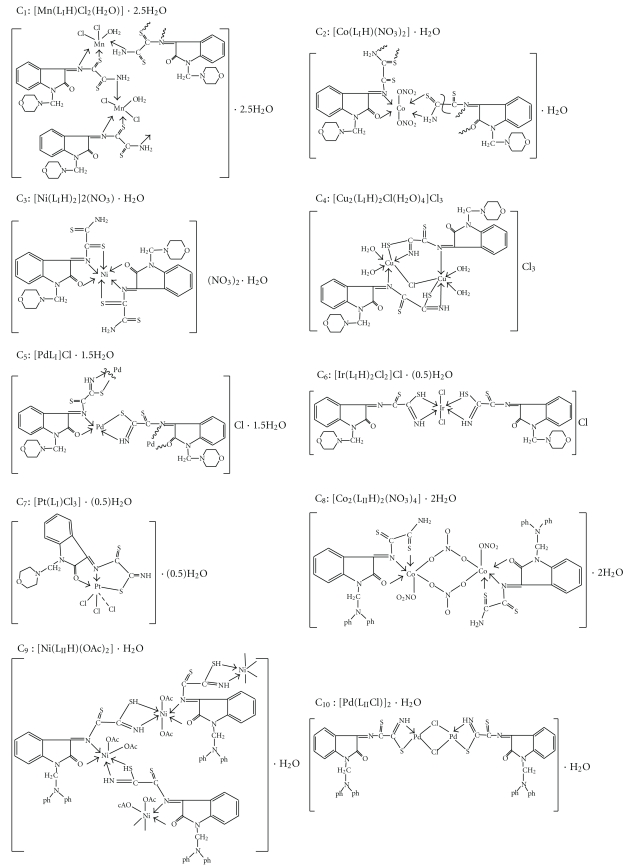

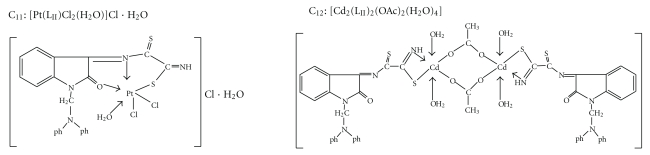
Suggested structures of Schiff and Mannich base complexes.

The spectrum of the Cd(II) complex (C_12_) exhibited charge transfer bands only, which is a common phenomenon for d^10^ metal complexes where d-d transitions are excluded [47, 48]. Conductivity measurement of metal complexes in DMF solution (10^−3^ M) showed nonelectrolytic nature of Mn(II), Co(II), and Pt(IV) complexes of L_I_H (C_1_, C_2_, and C_7_, resp.) and Co(II), Ni(II), Pd(II), and Cd(II) complexes of L_II_H (C_8_–C_10_ and C_12_, resp.) [50]. Electrolytic nature of 1 : 1 was exhibited by Pd(II), Ir(III) complexes of L_I_H (C_5_ and C_6_) and Pt(IV) complex of L_II_H (C_11_), 1 : 2 by Ni(II) complex of L_I_H (C_3_) and 1 : 3 by Cu(II) complex of L_I_H (C_4_) [50]. According to the above-mentioned data and those of elemental analyses and i.r. spectra, the structures of the metal complexes can be suggested as illustrated in Scheme 3.

## 7. Biological Activity

### 7.1. Antibacterial Activity

The growth inhibition of the prepared Schiff and Mannich base ligands and some selected metal complexes were studied against three types of pathogenic bacteria, namely, *Proteus mirabilis*, *Escherichia coli,* and *Staphylococcus aureus* by using DMSO as a solvent and the antibiotic Ceftriaxone as a control. Cultures were incubated at 37°C for 24 h. The inhibition zones were measured, and results are described in Table 5. The Schiff base precursor (SBH) and L_I_H were potent against all types of bacteria with the latter being more active than Ceftriaxone, while L_II_H was inactive. Complexes of L_I_H with Co(II), Ni(II), Pd(II), and Ir(III) ions (C_2_, C_3_, C_5_, C_6_) showed no activity while the Pt(IV) complex (C_7_) was as active as the original ligand against all types. Among the selected metal complexes of L_II_H, the Pd(II) complex (C_10_) was highly potent against all bacterial cultures. These results indicate that the degree of growth inhibition is highly dependent on the structure of ligands, metal complexes, and type of metal ion [51, 52]. Although the inhibition zones of L_I_H, C_7_ and C_10_  were larger than that caused by Ceftriaxone, other categories, like toxicity of these compounds, still have to be studied in detail.

### 7.2. Cytotoxic Effect

**Figure 9:**
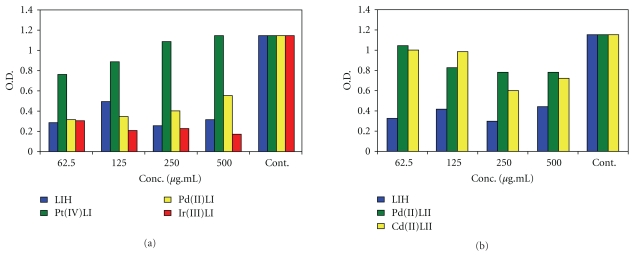
Cytotoxic effect of L_I_H, L_II_H, and some selected metal complexes on growth of cancer cell line Hep-2 at different concentrations with exposure time 48 h.

Preliminary cytotoxicity tests of the Schiff base (SBH) and its Mannich base ligands (L_I_H and L_II_H) with some selected metal complexes were performed in triplicate against cancer cell line of human epidermoid larynx carcinoma (Hep-2) using concentrations of 62.5, 125, 250, and 500 *μ*g/mL in DMSO with exposure time of 48 h using ELISA spectrophotometer. The three organic compounds showed high toxic activities at 125, 250, 250 *μ*g/mL, respectively, causing cell death as was confirmed by the drop in optical absorbance of NR in the treated cells compared with the controls which refers to complete disruption of cell functions [53]. The cytotoxic effect of metal complexes of L_I_H was found to increase in the order of Pt(IV) < Pd(II) << Ir(III) as is shown in Figure 9. The Pt(IV) complex (C_7_) was much less active than the parent ligand, and its performance was found to decrease with concentration that it was totally inactive at 500 *μ*g/mL.

**Figure 10:**
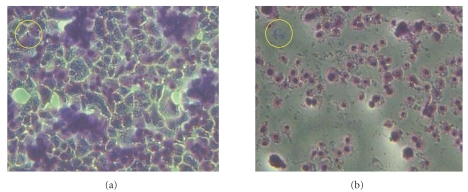
Tissue culture sections of Hep-2 cell line before (c) and after treatment with IrL_I_ (C_6_).

The Pd(II) complex (C_5_) followed the same trend of concentration as the Pt(IV) complex but it was toxic enough to cause cell death. The Ir(III) complex (C_6_) was exceptionally active, and its cytotoxic activity was found to increase with concentration.  Figure 10 illustrates the difference in dye distribution in the tissue culture sections of Hep-2 before and after treatment with this complex in comparison with the control. Both the Pd(II) and Cd(II) complexes of L_II_H (C_10_ and C_12_) were less toxic than the parent ligand, and their activity slightly increased with concentration. The Cd(II) complex (C_12_) caused 30–60% decrease of ligand activity. The complex was similarly inactive against growth of the three bacterial cultures although Cd^++^ ion is well known as an environmental carcinogen at very low concentrations in human and animals [54]. This indicates that the toxicity of Cd^++^ ion can be decreased on complexation with some ligands.

The present study describes a narrow scope of the cytotoxic activity of the studied compounds. Extension of this study, in future work to involve other cancer cell lines and other metal complexes, using normal human cell lines as control, may reveal more important data.

## 8. Conclusions

Condensation of dithiooxamide with isatin or its N-Mannich bases occurred from one amino end of the compound which allowed for tautomerism of the resulted compound in solutions as was confirmed by ^1^H.n.m.r spectrum of the product and from the IR spectra of some of its coordination compounds. The potent chelating behavior of the two new Mannich and Schiff bases led to the formation of bi- and polynuclear metal complexes. The preliminary study of biological activity showed some controversy in performance between bacterial growth inhibition and cytotoxic activities against Hep-2 cell line. The Ir(III) complex of L_I_H which showed the highest cytotoxic effect was almost inactive against bacterial growth.

## Figures and Tables

**Table 1:** Physical properties and analytical data of the prepared Schiff and Mannich base complexes.

Molecular formula (Color)	m.p. (decomposition) temp. °C	Yield %	% element analysis found (calculated)				
			C	H	N	M	Cl
[Mn(L_I_H)(H_2_O)Cl_2_] 2.5H_2_O (Brown) (C_1_)	215	42.24	33.91 (33.46)	4.22 (4.27)	11.18 (10.40)	10.66 (10.20)	13.11 (13.19)
[Co(L_I_H)(NO_3_)_2_]H_2_O (Brown) (C_2_)	174	61.11	33.11 (32.77)	3.02 (3.27)	16.09 (15.29)	10.50 (10.73)	—
[Ni(L_I_H)_2_]2NO_3_·H_2_O (Bright blue) (C_3_)	>300	50.8	40.90 (40.13)	3.62 (3.78)	16.06 (15.60)	5.78 (6.54)	—
[Cu_2_(L_I_H)_2_Cl(H_2_O)_4_]Cl_3_ (Reddish brown) (C_4_)	>300	77.11	40.25 (39.85)	4.32 (4.43)	6.81 (6.20)	13.73 (14.07)	7.26 (7.86)
[PdL_I_]Cl·1.5H_2_O (Dark brown) (C_5_)	250	59.21	34.45 (34.82)	3.01 (3.48)	11.32 (10.80)	—	7.01 (6.86)
[Ir(L_I_H)_2_Cl_2_]Cl·H_2_O (Pale yellow) (C_6_)	230	32.31	34.73 (35.51)	3.32 (3.55)	11.82 (11.05)	19.56 (19.12)	9.98 (10.50)
[Pt(L_I_)Cl_3_]0.5H_2_O (Yellow brown) (C_7_)	>300	34.01	27.78 (27.31)	2.49 (2.58)	8.82 (8.49)	28.89 (29.60)	16.82 (16.16)
[Co_2_(L_II_H)_2_(NO_3_)_4_]·2H_2_O (Dark green) (C_8_)	235	42.15	44.62 (43.74)	3.71 (3.17)	13.64 (13.31)	8.89 (9.34)	—
[Ni(L_II_H)(OAc)_2_] (Blue) (C_9_)	243	50.32	52.84 (53.40)	3.54 (3.95)	9.43 (9.23)	9.72 (9.23)	—
[PdL_II_Cl]_2_1.5H_2_O (Dark brown) (C_10_)	>280	23.30	46.51 (46.86)	3.81 (3.23)	10.11 (9.51)	—	6.40 (6.03)
[Pt(L_II_)Cl_2_·H_2_O]Cl·H_2_O (Brown) (C_11_)	250	33.23	36.21 (35.95)	2.13 (2.87)	8.23 (7.29)	24.77 (25.40)	13.09 (13.87)
[CdL_II_ (OAc)(H_2_O)_2_]_2_ (Yellow) (C_12_)	260	20.57	46.65 (47.06)	3.62 (3.29)	9.50 (8.77)	16.95 (17.63)	—

**Table 2:** Important I.R. vibrations (cm^−1^) for the two Mannich and Schiff base ligands and their metal complexes.

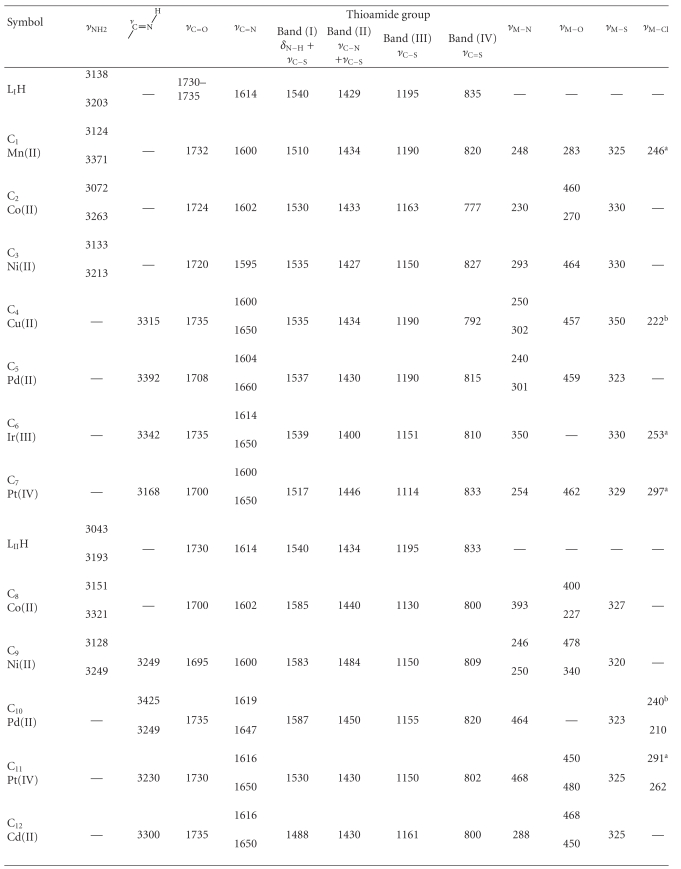

^a^: terminal; ^b^: bridging.

**Table 3:** Suggested thermal decomposition steps of C_2_ and C_6_.

Stable phase (M.wt) [CoL_**I**_ (NO_3_)_2_]·H_2_O (C_2_) (549.193)	Temp. range of decomp. at TG °C	Peak temp. at DTG °C	%weight loss found (calc.)
H_2_O (Lattice)	70–120	—	2.91 (3.27)
NO_3_	120–220	200	11.62 (11.97)
C_15_N_4_H_16_O_2_S_2_ NO_2_	220–600	—	71.38 (71.80)
CoO	—	—	13.28 (13.64)

[Ir(L_I_)_2_Cl_2_]Cl·(0.5)H_2_O (C_6_)(1004.24)			

0.5H_2_O (lattice) C_8_N_2_H_16_O_2_	80–250	190	17.43 (17.62)
3Cl C_9_N_2_H_8_	250–500	399	24.07 (24.94)
C_10_N_3_H_6_OS_2_	500–700	515	24.48 (24.69)
C_2_NS_2_H_2_	700–830	760	13.30 (13.14)
Ir	—	—	19.83 (19.12)

**Table 4:** Electronic spectra, spectral parameters, molar conductivity, and effective magnetic moments (*μ*_eff_) of Schiff and Mannich base complexes.

Comp. no.	Band positions (cm^−1^)	Assignment	Dq/B^/^(B^/^)(cm^−1^)	*β*	10Dq (cm^−1^)	Ω (S.mol^−^.cm^2^)	*μ*_ eff_ (BM)
C_1_ Mn(II)	*ν*_1 _17857	^6^A_1_g(S)→^ 4^T_2_g(G)	—	—	—	15.0	5.851
	*ν*_2_ 25641	L → M (C.T.)					
C_2_ Co(II)	*ν*_1_ 5352^(∗)^	^4^T_1_g →^ 4^T_2_g	0963(705)	0.726	6789	6.05	5.446
	*ν*_2_ 13333	^4^T_1_g (F) →^ 4^A_2_g					
	*ν*_3_ 16625	^4^T_1_g(F)→^ 4^T_1_g (P)					
	*ν*_4_ 27777	L → M (C.T.)					
C_3_ Ni(II)	*ν*_1_ 10204	^3^A_2_g →^ 3^T_2_g^3^	1.65(619.6)	0.602	10223	186.0	4.18
	*ν*_2_ 14388	A_2_g →^ 3^T_1_g (F)^3^					
	*ν*_3_ 21276	A_2_g →^ 3^T_1_g (P)					
C_4_ Cu(II)	*ν*_1_ 11111	^2^B_1_g →^ 2^A_1_g	—	—	—	282.0	2.51
	*ν*_2_ 16667	^2^B_1_g →^ 2^B_2_g					
	*ν*_3_ 22222	^2^B_1_g →^ 2^Eg					
	*ν*_4_ 28571	L → M (C.T.)					
C_5_ Pd(II)	*ν*_1_ 16393	^1^A_1_g →^ 1^A_2_g	—	—	—	77.0	Diamag.
	*ν*_2_ 21276	^1^A_1_g →^ 1^B_1_g					
C_6_ Ir(III)	*ν*_1_ 14705	^1^A_1_g →^ 3^T_1_g	—	—	—	90.0	Diamag.
	*ν*_2_ 18518	^1^A_1_g →^ 1^T_2_g					
	*ν*_3_ 22222	L → M (C.T.)					
C_7_ Pt(IV)	*ν*_1_ 15625	^1^A_1_g →^ 3^T_1_g	—	—	—	15.0	Diamag.
	*ν*_2_ 21276	^1^A_1_g →^ 3^T_2_g					
	*ν*_3_ 23255	L → M (C.T.)					
C_8_ Co(II)	*ν*_1_ 5471^(∗)^	^4^T_1_g →^ 4^T_2_g	0.843 (762.61)	0785	6430	18.0	4.617
	*ν*_2_ 10989	^4^T_1_g →^ 4^A_2_g					
	*ν*_3_ 15795	^4^T_1_g (F) →^ 4^T_1_g (P)					
	*ν*_4_ 26315	L → M (C.T.)					
C_9_ Ni(II)	*ν*_1_ 10172	^3^A_2_g →^ 3^T_2_g	1.667 (612.2)	0594	10200	20.5	4.251
	*ν*_2_ 14845	^3^A_2_g →^ 3^T_1_g (F)					
	*ν*_3_ 22727	^3^A_1_g →^ 3^T_1_g (P)					
	*ν*_4_ 27027	L → M (C.T.)					
C_10 _ Pd(II)	*ν*_1_ 16025	^1^A_1_g →^ 1^A_2_g	—	—	—	9.0	Diamag.
	*ν*_2_ 21739	^1^A_1_g →^ 1^B_1_g					
	*ν*_3_ 26315	^1^A_1_g →^ 1^Eg					
C_11_ Pt(IV)	*ν*_1_ 15908	^1^A_1_g →^ 3^T_1_g	—	—	—	73.0	Diamag.
	*ν*_2_ 27027	L → M (C.T.)					
C_12_ Cd(II)	*ν*_1_ 27777	L → M (C.T.)				8.00	Diamag.
	*ν*_2_ 32786	Intralig *π*→π*					

* Calculated.

**Table 5:** Antibacterial activities of the Schiff and Mannich bases and some selected metal complexes showing inhibition zones in diameters (mm).

Entry	Compound	*Proteus mirabilis*		*Escherichia coli*		*Staphylococcus aureus*	
1	SBH	19	++	30	+++	29	+++
2	L_I_H	32	++++	48	+++++	43	+++++
3	Co(II) (**C_2_**)	—	—	—	—	—	—
4	Pd(II) (**C_5_**)	—	—	8	—	9	—
5	Ir(III) (**C_6_**)	—	—	10	—	9	—
6	Pt(IV) (**C_7_**)	38	+++++	39	+++++	44	+++++
7	L_II_H	9	—	9	—	12	—
8	Co(II) (**C_8_**)	9	—	8	—	15	+
9	Pd(II) (**C_10_**)	38	+++++	39	+++++	44	+++++
10	Cd(II) (**C_12_**)	9	—	8	—	15	+
11	ceftriaxone	28	+++	30	+++	36	++++
